# Decreased expression of the satiety signal receptor CCKAR is responsible for increased growth and body weight during the domestication of chickens

**DOI:** 10.1152/ajpendo.00580.2012

**Published:** 2013-02-26

**Authors:** Ian C. Dunn, Simone L. Meddle, Peter W. Wilson, Chloe A. Wardle, Andy S. Law, Valerie R. Bishop, Camilla Hindar, Graeme W. Robertson, Dave W. Burt, Stephanie J. H. Ellison, David M. Morrice, Paul M. Hocking

**Affiliations:** University of Edinburgh, Roslin Institute and Royal (Dick) School of Veterinary Studies, Easter Bush, United Kingdom

**Keywords:** appetite, cholecystokinin type A receptor, chicken, domestication, agouti-related protein

## Abstract

Animal domestication has resulted in changes in growth and size. It has been suggested that this may have involved selection for differences in appetite. Divergent growth between chickens selected for egg laying or meat production is one such example. The neurons expressing AGRP and POMC in the basal hypothalamus are important components of appetite regulation, as are the satiety feedback pathways that carry information from the intestine, including CCK and its receptor CCKAR (CCK_1_ receptor). Using 16 generations of a cross between a fast and a relatively slow growing strain of chicken has identified a region on chromosome 4 downstream of the CCKAR gene, which is responsible for up to a 19% difference in body weight at 12 wk of age. Animals possessing the high-growth haplotype at the locus have lower expression of mRNA and immunoreactive CCKAR in the brain, intestine, and exocrine organs, which is correlated with increased levels of orexigenic AGRP in the hypothalamus. Animals with the high-growth haplotype are resistant to the anorectic effect of exogenously administered CCK, suggesting that their satiety set point has been altered. Comparison with traditional breeds shows that the high-growth haplotype has been present in the founders of modern meat-type strains and may have been selected early in domestication. This is the first dissection of the physiological consequences of a genetic locus for a quantitative trait that alters appetite and gives us an insight into the domestication of animals. This will allow elucidation of how differences in appetite occur in birds and also mammals.

it has been hypothesized that appetite has been modified as part of the domestication process to increase food intake and facilitate increased growth in birds (10, 17, 53) and mammals (49) in the pursuit of increased efficiency and rapid growth. Normally, appetite is regulated to ensure an optimal body weight and adiposity; however, there is clearly genetic variation in the “set point” for body weight (36), and different set points may be favored in different environments (45). The appetite control system includes central and peripheral systems (78). Activation of proopiomelanocortin (POMC)-containing neurons in the arcuate nucleus of the hypothalamus or its equivalent in birds results in weight loss through a reduction in appetite (9, 78); activation of agouti-related protein (AGRP)-containing neurons antagonizes the effect of POMC through the melanocortin MC4 receptor, and therefore, they are orexigenic or appetite promoting. A number of peripheral systems feed back to these neurons to determine the level of food intake and ultimately the growth of an animal. These include adiposity signals such as insulin, gut peptides such as cholecystokinin (CCK), and peptide YY and the afferent vagus (78). In mammals leptin participates in the feedback, but in the chicken this system is absent (66), and the action of ghrelin on food intake has been shown to be the opposite of that found in mammals (25).

Utilizing a cross of fast- and relatively slow-growing chicken strains, it has been possible to map genetic loci [quantitative trait loci (QTL)] for growth characteristics (32, 65), the most significant of which is on chromosome 4. Using an advanced intercross between a fast-growing broiler strain and a relatively slow-growing egg laying strain, we have narrowed down the region and identified the expression of the CCK type A receptor (CCKAR) gene as the candidate for differences in growth at the locus on chromosome 4. In mammals, CCK acts predominately on the G protein-coupled receptor CCKAR (HUGO and Chicken Gene Nomenclature Consortium; http://www.agnc.msstate.edu/), also known as the CCK_1_ receptor (IUPHAR nomenclature), to reduce food intake and increase satiety (67). In birds, although it is well established that exogenous CCK inhibits short-term food intake (61), experiments with CCK receptor antagonists have been inconclusive (55). Our study provides new evidence for the location of CCKAR within the avian brain and shows that CCKAR is less abundant in birds carrying the high-growth allele. Peripheral signaling of satiety is altered between the genotypes, resulting in reduced sensitivity to the CCK satiety signal. In turn, the central levels of expression of the hypothalamic orexigenic peptide AGRP are increased in birds carrying the high-growth allele.

## METHODS

### Animal Populations

#### Advanced intercross population (advanced intercross line).

Offspring from a reciprocal mating of a White Leghorn, which is a light-layer line, and a commercial male line of broilers, which is a heavy-meat chicken, were used to establish an advanced intercross line (AIL) to allow fine mapping (14). For the F2 onward, five males and five females were selected from 12 families to minimize inbreeding (22). Female offspring in the F8 and offspring from both sexes in the F16 were reared in floor pens (3 × 2 m) on ad libitum-layer rations on an 8:16-h (light-dark) photo period. Body weight was recorded at 3, 6, 9, 12, and 20 wk of age.

Four male and 11 female F16 parents heterozygous for the CCKAR haplotype (see *Genotyping*) were mated to avoid brother-sister matings, and the offspring were reared in floor pens on a 14:10-h (light-dark) photo period. They were transferred to individual cages at 2 wk of age and fed ad libitum on a standard-layer starter diet.

#### Multistrain.

The multistrain population is not a single population, as the name suggests, but a set of samples from a large range of chicken strains. It comprised males of *1*) 13 traditional or pure breeds with a range of growth phenotypes (Auracana, Barnevelder, Brown Leghorn, Buff Orpington, Cornish Game, Friesian Fowl, Ixworth, Jersey Giant, J-line, Light Sussex, Maran, White Dorking, White Sussex), *2*) 12 modern-meat type lines, and *3*) 12 modern layer lines. The populations have been described previously (58). Twelve individuals of each breed sired by two to four males were available with records of body weight at 10 wk of age that were used to compare genotypes with phenotypes.

Animal care and use protocols were approved by the Roslin Institute. All animal work was carried out under the UK Home Office license guidelines and regulations [Animal Scientific Procedures Act (1986)].

### Genotyping

Informative single nucleotide polymorphisms (SNP) on chromosome 4 were selected using a SNP finder (74) and targeted sequencing and from testing Beijing SNPs (76) on the founders of the AIL using a SNPlex assay.

Genotyping was carried out using a SNPlex assay (Applied Biosystems, Paisley, UK) and checked using a genotype checker (52) for pedigree and genotype quality. Markers and individuals were removed if there was >20% error. The 77 successful genotypes between 1,759,990 and 88,895,200 on chromosome 4 were managed and stored using resSpecies (38). All positions in this article are referenced to the 2006 build of the genome. Genotyping of markers ch4snp-131-42-9157-S-2, ch4snp-85-282-3484-S-1, ch4snp-31-245-11611-S-1, ch4snp-131-132-4046-S-2, ch4snp-85-157-3063-S-2, and CCKAR_MnlI for estimation of association in generation F16 and in the multistrain animals was carried out by Kbiosciences (Hoddesdon, Herts, UK). These markers were all completely informative in the AIL. In other words, they were fixed in the founders, except for ch4snp-85-282-3484-S1 and ch4snp-131-42-9157-S2, which were not fully informative; i.e, in one of the lines both alleles were found.

### Genotyping of Markers in the CCKAR Gene for Selection of Experimental Birds

A SNP marker, CCKAR_MnlI, in exon 3 of the CCKAR gene was chosen to represent the CCKAR haplotype, differentiating the AIL founders combined with the most significant marker flanking the CCKAR gene, ch4snp-131-132-4046-S-2, to produce a larger haplotype for selection. CCKAR_MnlI was genotyped using a *Mnl*I RFLP using primers CCKAR_F3 and CCKAR_altR3 (Table 1), ch4snp-131-132-4046-S-2 by an *Alu*I RFLP (G/T) using Chr4:77192329F and Chr4:77192329R primers (Table 1), and ch4snp-32-183-65476-S-1 by a *Mwo*I RFLP (A/G) using Chr4:65117463F and Chr4:65117463R primers (Table 1).

**Table 1.** Primers used in the sequencing, real-time PCR, or genotyping of the CCKAR gene loci

CCKAR exon1F	GTGGTGTTGGTTGAGAGACG
CCKAR exon1R	CAGCAAAGCAGTGATGTTGG
CCKAR exon1Fb	AACCAGCCTTCTTCCTAGCAG
CCKAR exon1Rb	GTGCACTGCTCATTCACCAC
CCKAR exon2F	GGCTTTTGAAAGGGTGTACCT
CCKAR exon2R	TTCTCACATACCCCACTGGTT
CCKAR_F3	CATTTGAAAACAGCAGAAGCA
CCKAR_altR3	CTGCTGAATGACATCACTTGG
CCKAR exon3Rb	TTACAGCAGGGGCTTAGCAG
CCKAR exon4F	AGGCACTCCCCTTTAAGAGC
CCKAR exon4R	GGTCCTAATCCACAGCCAGA
CCKAR exon5F	GGCCAAGAAACTTGTCATCC
CCKAR exon5R	CTCACAGCGTTTACTGTCAGC
CCKAR exon5Fb	AATACTGGTGGCCAGCAAAC
CCKAR exon5Rb	CTCTAATCACAGGCGGCTTC
CCKAR 3′ F	GTTTCCGCATGGGTTTTCTA
CCKAR 3′ R	TTCAGCCTTATCCCTGTGCT
CCKAR upstreamF	TACCCTTGAGGCTGGAAATG
CCKAR upstreamR	TCTCAGAGGGAGGTTGCTGT
Chr4: 77192329F	TCCATGGACCTCTTATCCTCA
Chr4: 77192329R	TGCTCTCATCCAATGACACAT
chr4: 65117463F	CGGCGATACTTCGTAGCACT
chr4: 65117463R	CTCAGCTGTATCCCAGCACA
CCKAR ex-F2	TACAGCAAGCTGGTCCCTTT
CCKAR ex-R2	AATGAAATGAGGCCATACGC
RBPJ_F1	CCATGCCAGTTCACAACAGT
RBPJ_R1	CGGATCATCTGCATCCAATA
Slc34a2_F1	TGAAGATGCCCCTGAGCTAC
Slc34a2_R1	GGTCCACGTCACATTTCCTT
PI4K2B_F1	TGGTTCTTCCTCGCATCTCT
PI4K2B_R1	GTGGCATCTGAACAAGCTGA
SEPSECS_F1	GTTGGCAGACATCCACAATG
SEPSECS_R1	GCAGCATTGAGGTAAGCACA
SOD3_F1	GTTGTGTCCGATCCCACCT
SOD3_R1	TGAAGTAACACGCTGCTTGG
DHX15_F1	GATCTGGGCGACGATTACAG
DHX15_R1	TGTGTGGAATCATTGCTGGT
PPARGC1A_F1	ACAAAACCATGCGAACACAA
PPARGC1A_R1	TTGGTCTGAGGAGGGTCATC
LCORL_F1	AGGGCTTTATGGACCAAGGC
LCORL_R1	CTGACGCACGATCACTGACT
SLIT2_F2	TGGAGCCTCTGGTGTCAATG
SLIT2_R2	CGGTGGTGATCTGGTTGTCATA

CCKAR, CCK type A receptor.

### Analysis

Screening of the data for association was carried out using GenAbel (1). The most significant markers were subsequently fitted by restricted maximum likelihood (Genstat Version 12), including pen and sire, using a linear model as described previously (21), followed by approximate Student *t*-tests to assess marker effects. In the F16 generation, sex was also included in the model. The additive effect of each marker was estimated as one-half of the difference between homozygote means.

### Sequencing of CCKAR

Primers (Table 1) were designed to flank all exons, the putative proximal promoter, and the region downstream of the CCKAR gene using Primer 3 software (56). Resequencing was carried out at the CCKAR locus from the 16th generation of the advanced intercross line, four individuals from the high- and four from the low-growth genotype, by GATC-Biotech (Konstanz, Germany).

### Effect of CCK Injection on Food Intake

Eight individually housed animals of each CCKAR genotype were handled daily for 1 wk prior to injection. At 3 wk of age, 0 or 10 μg/kg sulfated CCK octapeptide (Tocris Bioscience, Bristol, UK) was administered via an intraperitoneal (ip) injection, with 1 day between the randomized doses. This is in the middle of the dose response range (8, 12). Each bird acted as its own control. Food intake was recorded at 30 min after injection. The birds were returned to their home pens, and at 12 wk of age the birds were euthanized by barbiturate overdose. The basal hypothalamus, preoptic area of the hypothalamus, pituitary, hindbrain, duodenum, small intestine, ceca, gall bladder, and pancreas were collected and immediately frozen in liquid nitrogen and stored at −80°C. The basal and preoptic area of the hypothalamus dissection has been described previously (37); the duodenum sample was taken from the tip of the pancreatic loop and the small intestine 1 cm posterior to the junction of the ceca. The samples from the same animals were also used for estimation of allele-specific expression.

### Expression Analysis

#### RNA isolation and reverse transcription.

Total RNA was extracted from <100 mg of tissue using Ultraspec II reagent (AMS Biotechnology, Abingdon, UK) and Lysing Matrix D tubes in a FastPrep Instrument (MP Biomedicals, Cambridge, UK) or homogenized using a T10 Ultraturrax (IKA Werke), depending on tissue type. Total RNA was reverse transcribed using NotI-(dT)18 primer and a First-Strand cDNA synthesis kit (GE Healthcare, Buckinghamshire, UK) according to the manufacturer's protocol. cDNA samples were diluted ×15 for use in real-time PCR.

#### Real-time PCR assays.

CCKAR-, AGRP-, and LBr-specific primers were designed using Primer 3 (56) to amplify products of ∼100–200 bp in length crossing intron/exon boundaries, (Table 1). Similarly, assays were designed for genes in the vicinity of CCKAR and the markers most significantly associated with body weight, i.e., RBPJ (chr4: 75,648,820), Slc34a2 (chr4: 75,884,660), PI4K2B (chr4: 75,987,346), SEPSECS (chr4: 76,012,821), SOD3 (chr4: 76,176,057), DHX15 (chr4: 76,221,629), PPARGC1A (chr4: 76,629,533), SLIT2 (chr4: 77,708,527), and LCORL (chr4:78,711,367) (for primers used, see Table 1). PCR products for standards were purified using a QIAEX II gel extraction kit (Qiagen, Crawley, West Sussex, UK) and their concentrations measured using a NanoDrop spectrophotometer (Thermo Fisher Scientific, Loughborough, Leicestershire, UK). Serial dilutions of standards were made to create standard curves for real-time PCR quantification. Real-time PCR reactions were run on an MX3000p real-time PCR machine (Agilent Technologies, Cheshire, UK) using the following conditions: 95°C for 2 min, 40 cycles of 95°C for 15 s, and 60°C for 30 s. Real-time PCR reactions (25 μl) were run using a 10-μl cDNA template together with SYBR green master mix (VHBio Ltd, Gateshead, UK) and gene-specific primers (100 nM). Samples and standard curves were run in duplicate on the same 96-well plate along with water blank controls. Standards were diluted to produce top standards detectable after ∼15 PCR cycles. Assays were analyzed using MxPro software (Agilent Technologies), and both CCKAR and AGRP expression were normalized using lamin B receptor expression (42).

#### Allele-specific expression.

To determine whether the slow- and high-growth alleles were differentially expressed in heterozygotes, we utilized one of the synonymous SNPs (dbSNP ss 550122602) that segregated between the genotypes and was present in exon 5 of the CCKAR gene. The SNP could be diagnosed by digestion with restriction enzyme *Acu*I on hypothalamic cDNA amplified using primer CCKARex-F2 and CCKARexon5Rb, which produced sizes of 435 and 220 bp for the high-growth allele and 655 bp for the low-growth allele. Digestion was carried out using 10 μl of PCR product, 1 μl of buffer 4 (New England Biolabs), 0.025 μl of 32 mM *S*-adenosylmethionine, 8.775 μl of H_2_O and 1 unit of *Acu*I (0.2 μl) per sample and incubated for 1 h at 37°C. Products were run on a 2% agarose gel containing sybrsafe (Invitrogen, Paisley, UK). The intensity of the bands was calculated using Image J (http://imagej.nih.gov/ij/) on images taken using a G:Box imager (Syngene, Cambridge, UK). The sum of the intensity of the 435- and 220-bp bands was compared with the 655-bp band intensity using a paired *t*-test.

### Immunocytochemistry

Three peptide epitopes from chicken CCKAR (CYLHKAKRKRKVPLQQ, LATFTCCAKQKPPVIRG, and RAFDTASADLHLSGAP) were conjugated to KLH and used to immunize two rabbits by Dundee Cell Products (Dundee, UK). Antibody titers of anti-CCKAR in three bleeds from the two rabbits were measured by an ELISA using the peptides used in raising the antibodies to coat the plates. The final bleed from rabbit 2 (CCKAR2F) was found to have the highest antibody titer (data not shown) and was used for immunohistochemistry.

At 12 wk of age, high-growth (*n* = 5 males and 2 females) and low-growth (*n* = 5 males and 3 females) haplotype birds were euthanized by a barbiturate overdose. Brains were removed and immediately immersed in 4% paraformaldehyde (wt/vol) in 0.1 M phosphate-buffered saline (PBS) solution (pH 7.4) for 5 days to fix the tissue. Brains were then transferred to 15% sucrose in 4% PFA for 48 h and to 30% sucrose in 0.1 M PBS for a further 48 h. All incubations were carried out at 4°C. Brains were removed from the sucrose solution, with excess solution blotted with tissue paper, and snap-frozen on dry ice before coronal sections (60 μm) were cut on a freezing sledge microtome [from the tractus septomesencephalicus (TSM) to the level of the median eminence]. From preliminary investigations using this antibody, no evidence of positive cytoplasmic staining was found in sections anterior or posterior to these landmarks. Every second section was collected into 0.1 M PBS in a 12-well plate and stored at 4°C until processing. Free-floating sections were processed for CCKAR immunoreactivity using the CCKAR2F anti-serum diluted at 1:1,000 in 0.1 M PBS, 0.2% Triton-X-100 (vol/vol), and 3% normal goat serum (vol/vol) and incubated at room temperature for 2 h on a shaking platform before overnight incubation at 4°C. The antibody-antigen complex was visualized using a standard ABC protocol (43). CCKAR immunoreactivity was visualized using a Vectastain Elite kit (Vector Laboratories, Peterborough, UK), with 0.025% diaminobenzidine (wt/vol) as the chromogen and 0.03% hydrogen peroxide (vol/vol). Sections were mounted onto gelatin-coated slides, air-dried, and coverslipped with Pertex mounting medium (Leica Microsystems, Milton Keynes, UK). Omission of the primary antibody, blocking using the CCKAR peptide, or incubation with CCKAR preimmune serum resulted in a total lack of labeling (data not shown).

The total number of CCKAR-labeled cells in the hypothalamus was counted for each bird by using a light microscope under bright-field illumination (mean no. of sections analyzed: low-growth haplotype = 20.8; high-growth haplotype = 21.3). Throughout quantification the analyst was unaware of which group each brain belonged to.

## RESULTS

**Fig. 1.**
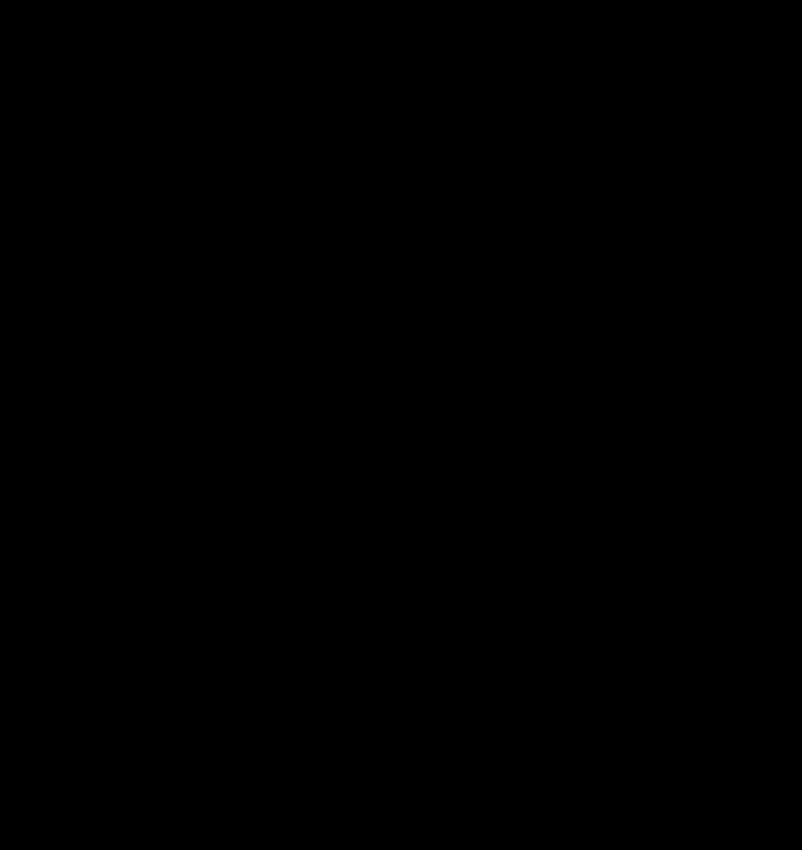
Significance (−log P) of association of markers along chromosome 4, with body weight at 12 wk of age in the F8 generation of the advanced intercross line using GenAbel (1). Pen was included as a factor. Each dot represents a single nucleotide polymorphism.

**Fig. 2.**
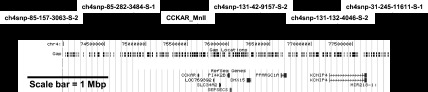
Position of the markers genotyped in the vicinity of cholecystokinin type A receptor (CCKAR) are indicated with an arrow and their name. Underneath is the genome map from the 2006 build from the University of California Santa Cruz browser (71a) showing distance in bp and the position of refseq genes. In the F16 generation, ch4snp-131-132-4046-S-2 and CCKAR_MnlI were the most consistently highly associated markers.

Dense genotyping of chromosome 4 identified SNPs with strong association with body weight and growth in the F8 AIL generation (Fig. 1). The SNPs with the most significant *P* values were close to the CCKAR, also known as the CCK_1_ receptor, in the IUPHAR nomenclature or CCKAR in the Chicken Gene Nomenclature Consortium nomenclature (http://www.agnc.msstate.edu/). The highest scoring marker, ch4snp-131-132-4046-S-2, is at position 77,192,329, which is 1.56 Mbp downstream of the CCKAR locus (Table 2 and Fig. 2).

**Table 2.** Significance of association (−logP) and size of the additive effect (AA-aa/2) of body weight (g) for each of the top-scoring SNPs at 3, 6, 9, and 12 wk of age in the F8 and F16 generation of the broiler layer advanced intercross

		Age, wk							
		3		6		9		12	
Marker	Position*	F8	F16	F8	F16	F8	F16	F8	F16
Significance of association (−logP)									
ch4 snp-85-157-3063-S2	73855056	0.22	5.06	1.62	2.70	3.78	2.15	3.68	1.89
ch4 snp-85-282-3484-S1	75174977	0.55	3.67	1.64	2.22	3.72	1.62	2.85	1.37
CCKAR_MnlI	75633208	0.40	4.19	2.05	3.77	4.38	2.70	3.44	2.40
ch4 snp-131-42-9157-S2	76180551	0.15	3.39	1.89	3.51	4.23	2.30	3.36	2.00
ch4 snp-131-132-4046-S2	77192329	0.83	4.66	4.24	5.25	7.40	4.51	6.43	3.69
ch4 snp-31-245-11611-S1	79811791	0.53	1.40	2.30	3.05	4.78	2.22	2.05	1.66
Body weight, g									
ch4 snp-85-157-3063-S2	73855056	1.8	18.1	21.2	48.7	61.5	83.0	82.5	139.5
ch4 snp-85-282-3484-S1	75174977	4.1	16.7	23.4	42.1	67.5	68.0	78.0	112.0
CCKAR_MnlI	75633208	2.8	18.1	23.5	57.0	64.0	93.5	76.0	159.5
ch4 snp131-42-9157-S2	76180551	1.4	16.9	24.4	58.0	69.0	90.0	83.0	151.0
ch4 snp131-132-4046-S2	77192329	4.7	21.3	35.7	56.6	84.5	139.5	106.5	230.0
ch4 snp31-245-11611-S1	79811791	3.8	10.6	27.9	58.1	74.0	93.0	61.5	144.5

SNP, single nucleotide polymorphism. The F8 generation was composed of female birds, whereas the F16 generation was of both sexes.

* The position is given on chromosome 4 of the 2006 build of the chicken genome.

An additional line-specific marker in the CCKAR gene, CCKAR_MnlI, was genotyped in the AIL F8 (∼350 animals with complete genotypes) and subsequently in the F16 generation (∼204 animals), and the data were reanalyzed using REML (Table 2) for the top markers from the GenAbel analysis. The estimate of the *P* value and the additive effect for each generation are shown in Table 2. The −log *P* values are highest for association in the F8 for 9-wk body weight, reaching 7.40 for ch4snp-131-132-4046-S-2. In the F16 generation, the −log *P* values are highest for association between ch4snp-131-132-4046-S-2 and body weight at 6 wk of age. ch4snp-131-132-4046-S-2 is the top-ranked marker, and CCKAR_MnlI is the second-ranked marker at all ages and generations, although association strength differs across time in the different generations, with no statistical association in the F8 at 3 wk of age and limited association in the F16 at 12 wk of age. The additive effect for body weight is correlated with the level of significance and is up to twofold larger in the F16 generation, reaching 230 g at 12 wk of age. The F16 population was of mixed sex, which explains the larger effect since males grow faster and are larger than females. *P* values at 1 wk of age did not pass a Bonferroni-corrected *P* value of 6.5 × 10^−04^ for 78 genotypes (data not shown). The QTL effect expressed as a percentage of the total variance using the reduction in the sum of the squares after the addition of the marker in the fitted model varies between 2.3 and 4.9% in the F8 and between 4.4 and 4.8% in the F16 depending on age.

To present this in a different way, the body weight (g) of females using the ch4snp-131-132-4046-S-2 marker as a classifier in the F8 population at 12 wk of age was as follows: GG: 2,406 ± 28, *n* = 92; GT: 2,319 ± 17, *n* = 223; TT: 2,258 ± 23, *n* = 120 (ANOVA, *P* < 0.001). Body weight of females in the F16 population at 12 wk of age was as follows: GG: 2,272 ± 57, *n* = 23; GT: 2,076 ± 29, *n* = 40; TT: 1,927 ± 37, *n* = 40 (ANOVA, *P* < 0.001). Body weight of males in the F16 population at 12 wk of age was as follows: GG: 2,951 ± 65, *n* = 23; GT: 2,664 ± 41, *n* = 60; TT: 2,546 ± 49, *n* = 46 (ANOVA *P* < 0.001). The difference represents differences in body weight of 6.5 to 19% between the TT and GG genotypes.

**Fig. 3.**
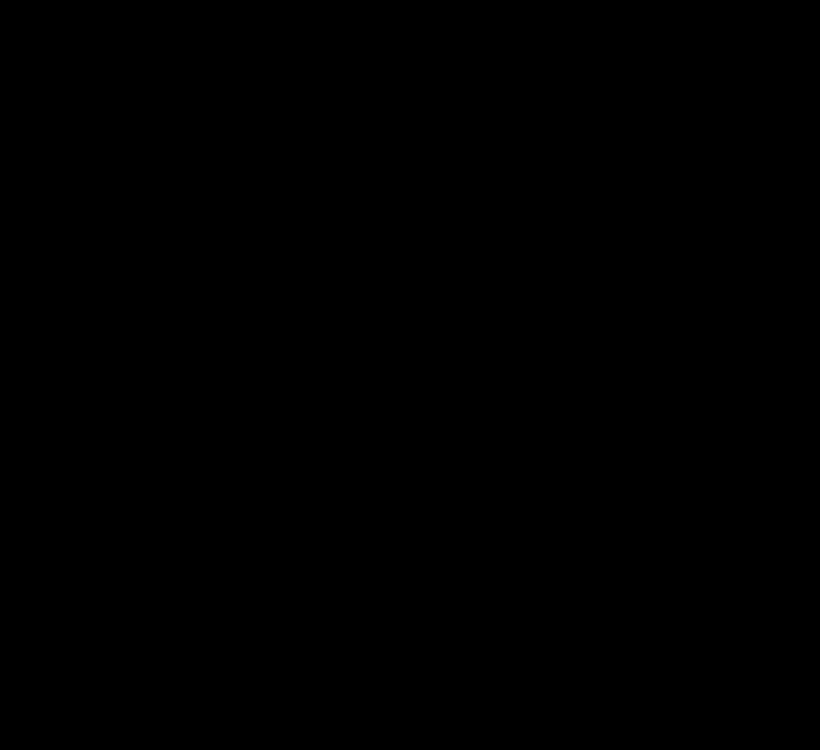
The prevalence of the CCKAR_MnlI CC (high-growth) allele in populations of traditional breeds (●) and modern commercial meat- (○) and layer-type chickens (▼) plotted against 10-wk mean body weight. Each population was comprised of 12 individuals with 24 alleles. All of the birds were housed in pens and fed ad libitum. The *inset* presents data for traditional and layer strains after removal of the meat-type strains, which are selected for high growth. The *r*^2^ from the regression analysis of the means is presented for this reduced data set.

It was hypothesized that if the CCKAR gene locus was responsible for variation in body weight, then alleles of the gene might be distributed differentially across strains of chicken with different growth phenotypes. Using the CCKAR_MnlI marker, which is in the gene itself, there was a clear relationship between the frequency of the marker for the high-growth allele and body weight at 10 wk of age (Fig. 3). There are some exceptions among the broiler strains, but the majority have a high frequency of the allele. The relationship is more evident when the broiler strains are removed (see Fig. 3, *inset*). The regression analysis indicated a correlation coefficient of 0.57 between the body weight of the reduced data set and the allele frequency in the population (*P* < 0.0001). Repeating this exercise with the other markers in Table 2 showed significant or nearly significant correlations only with ch4snp-31-245-11611-S-1 (*P* = 0.01) and ch4snp-85-157-3063-S-2 (*P* = 0.08).

### Selective Breeding for Sequencing, Physiology, and Expression

Specific characterization of the CCKAR gene was based on individuals produced by the mating of heterozygotes at a three-loci haplotype covering the region of the CCKAR gene ch4snp-32-183-65476-S-1, CCKAR_MnlI, and ch4snp-131-132-4046-S-2.

Sequencing of the CCKAR locus was completed for four homozygote offspring. The region sequenced flanked exon 1 and upstream (Chr4: 75,628,867–75,630,758 bp), exon 2 and flanking region (75,630,998–75,631,348 bp), exon 3 and flanking region (75,633,120–75,633,647 bp), exon 4 and flanking region (75,634,455–75,635,076 bp), and the region flanking exon 5 and the 3′ region (75,635,268–75,636,796 bp). The 4,917-bp sequence contained 36 variations, of which three were insertion deletions and two were known SNPs (snp.13.786.12116.S.3, snp.13.786.5607.S.1). We found no mutations associated with an amino acid change, splicing, or differences in the 5′ and 3′ end of the gene that was potentially able to effect gross expression. All 36 differences formed two distinct haplotypes. All SNP information and allele frequencies have been submitted to dbSNP with NCBI submitter SNP (ss) accession no. 550122***, where *** is 057, 082, 118, 147, 175, 200, 225, 249, 278, 304, 325, 352, 369, 405, 430, 455, 478, 501, 528, 556, 583, 602, 625, 649, 666, 691, 721, 745, 774, 799, 830, 855, and 890.

**Fig. 4.**
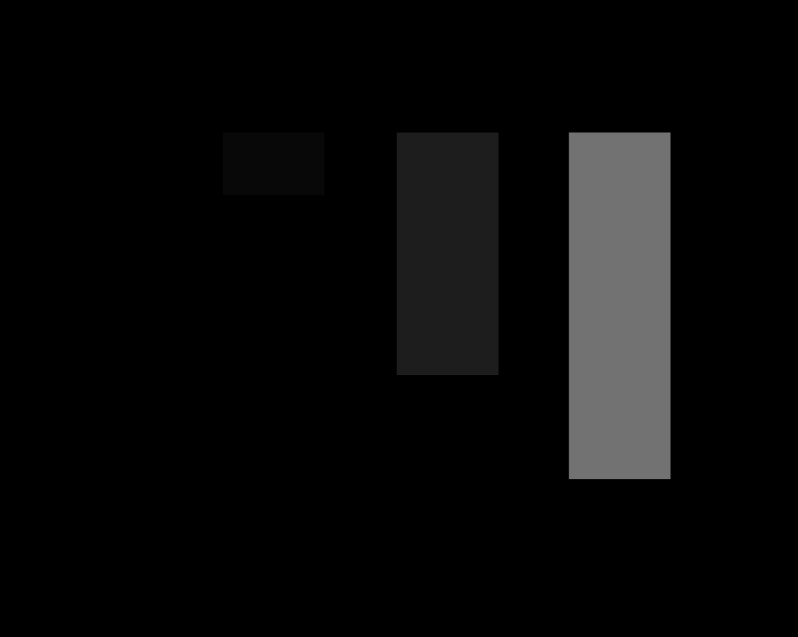
Difference in food intake 30 min after ip injection of CCK in homozygous high- or low-growth and heterozygote haplotypes. Each bird received either 0 or 10 μg of sulfated CCK octapeptide/kg body wt, with 1 day between the randomized doses. Data presented represent the mean ± SE of the difference between the food intake in the 30-min period after injection of the 0 and 10 μg CCK8/kg doses in individual animals (*n* = 8). The ANOVA *P* value is shown.

All of the evidence suggested that the CCKAR locus controlled growth rate and was responsible for an ≤19% difference in body weight between genotypes. One mechanism for this association was by altering the response to CCK centrally and peripherally. This hypothesis was tested by comparing the food intake of chickens to ip injection of CCK. This showed that the high-growth haplotype showed minimal response to CCK, whereas the low-growth haplotypes reduced their intake by 81% over the test period (Fig. 4).

**Fig. 5.**
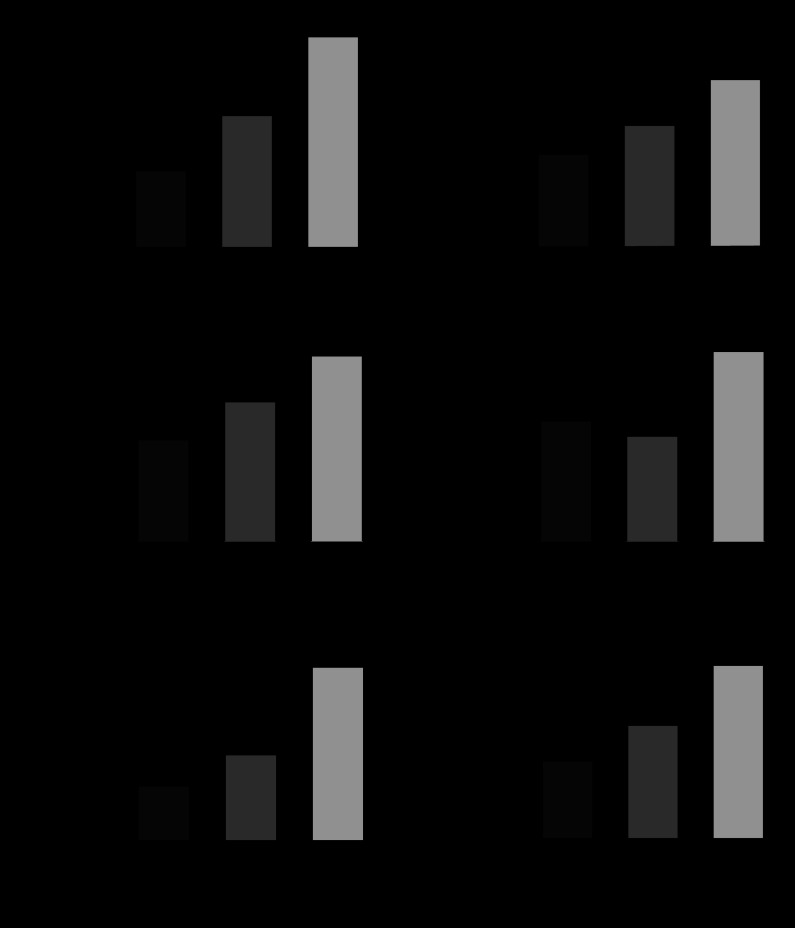
Expression (±SE) of CCKAR mRNA in tissues taken from 12-wk-old homozygous high- or low-growth and heterozygote (Het) haplotypes (*n* = 8/group). *P* values from the ANOVA on log-transformed data are given in each graph.

In the absence of mutations that affected the receptor structure, we examined whether CCKAR mRNA expression could explain the effect on growth and response to CCK injection. Levels of CCKAR mRNA at 12 wk of age were lower in the high-growth haplotypes than in the low-growth haplotype, with the heterozygotes usually intermediate. This was statistically significant in the duodenum, cecum, pancreas, hindbrain, and basal hypothalamus (Fig. 5), whereas the levels in the pituitary were similar in all genotypes. The magnitude of the difference was ∼1.6 in the duodenum and 3.1 in the cecum, with most around twofold. Expression of the receptor was highest in the pancreas, as expected (48), at ∼35-fold higher than in the cecum. Expression in the cecum, pituitary, and gall bladder was ∼10-fold more than the duodenum, hindbrain, and basal hypothalamus (Fig. 5).

Digestion of CCKAR cDNA from the hypothalamus to determine allele-specific expression was made by comparing the sum of the 435- and 220-bp bands for the high-growth allele and the 655-bp band corresponding to the low-growth allele by a paired *t*-test. This indicated that in the heterozygotes the difference between the band intensities was significantly different from zero (*P* < 0.009), with the ratio of the 655-bp band corresponding to the low-growth allele and the 435- and 220-bp bands from the high-growth allele being 3.54 ± 0.8 (low-growth allele/high-growth allele).

In case the genetic cause underlying the difference between the genotypes affected other genes in the locus, the expression of a number of genes in the vicinity of CCKAR and the markers with the highest association were examined. We did not find assayable expression for RBPJ, Slc34a2, DHX15, or PPARGC1A in the tissue panel we had available; however, LCORL, SLIT2, SOD3, PI4K2B, and SEPSECS showed no difference in expression between genotypes.

**Fig. 6.**
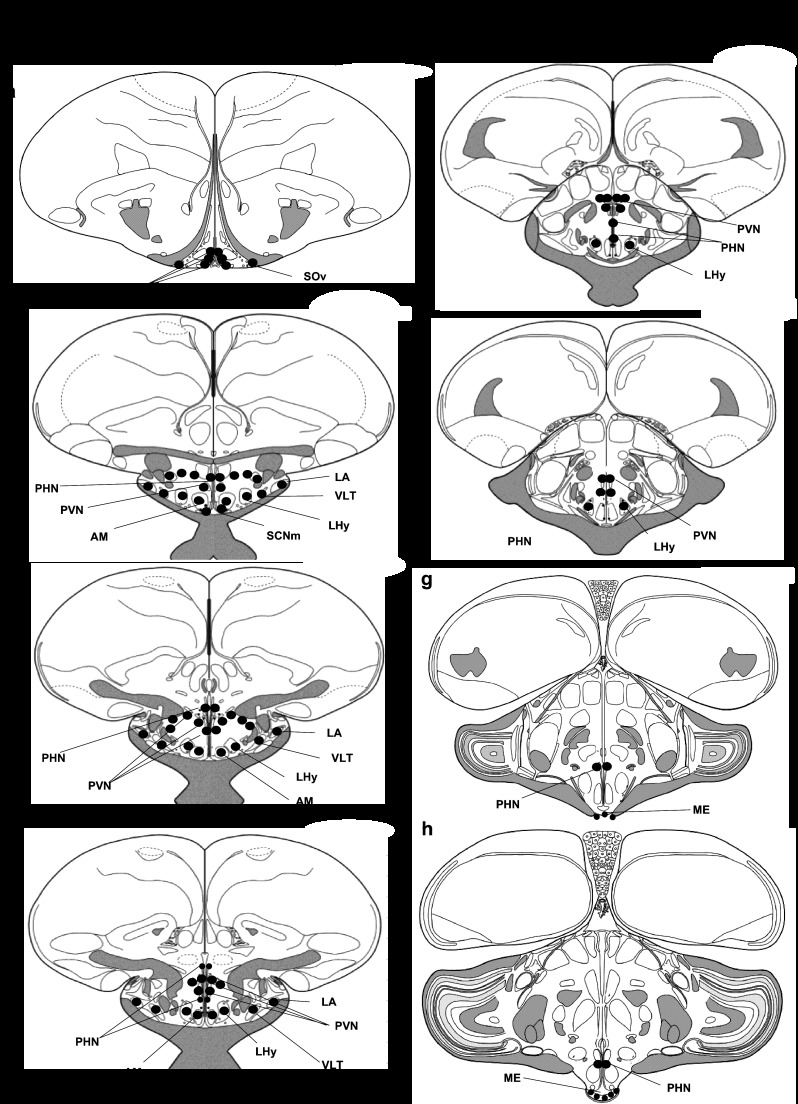

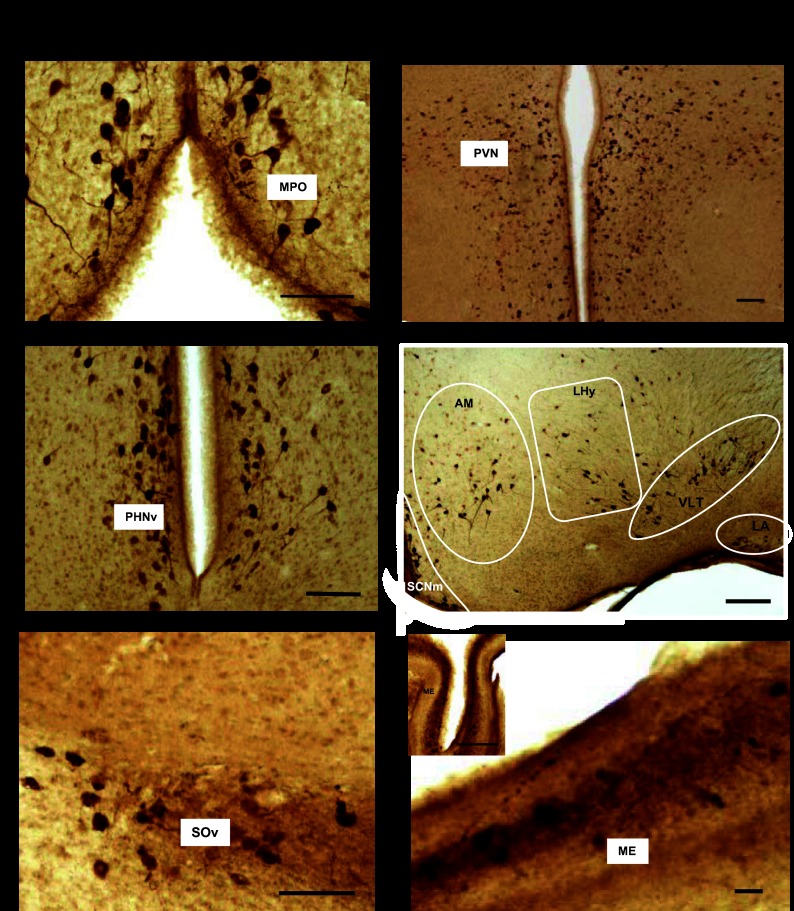
*A*: coronal rostrocaudal sections (*a*–*h*) showing anatomic distribution of CCKAR immunoreactivity in the hypothalamus of broiler chickens, represented by black dots. Atlas adapted from Prof. Wayne Kuenzel (http://avianbrain.org/nomen/Chicken_Atlas.html) (36a). *B*: localization of CCKAR immunoreactivity within the chicken hypothalamus. *Image a*: magnocellularis preopticus (MPO). *Image b*: paraventricular nucleus (PVN). *Image c*: ventral periventricularis hypothalamic (PHNv). *Image d*: nucleus suprachiasmaticus pars medialis (SCNm), anterior medialis hypothalami (AM), lateralis hypothalami (LHy), nucleus ventrolateralis thalami (VLT), and lateralis anterior thalami (LA). *Image e*: supraopticus pars ventralis (SOv). *Image f*: fiber tracts and processes within the median eminence (ME). All scale bars, including *image f*, *inset*, represent 10 μm. Scale bar in *image F* (main image) represents 1 μm.

Using immunohistochemistry, CCKAR immunoreactivity was found to be widely distributed in the brain of the chicken, with perikarya observed from the level of the TSM to the median eminence (see Fig. 6*A*).

Most hypothalamic brain regions contained CCKAR-immunoreactive nerve fibers. A dense innervation was observed in the paraventricular nucleus (PVN) and median eminence (ME). CCKAR-immunoreactive perikarya were observed in the supraopticus pars ventralis, magnocellularis preopticus, periventricularis hypothalamic (PHN), PVN, anterior medialis hypothalami (AM), region lateralis hypothalami, nucleus ventrolateralis thalami, nucleus lateralis anterior thalami, and nucleus suprachiasmaticus, pars medialis (see Fig. 6, *A* and *B*).

**Fig. 7.**
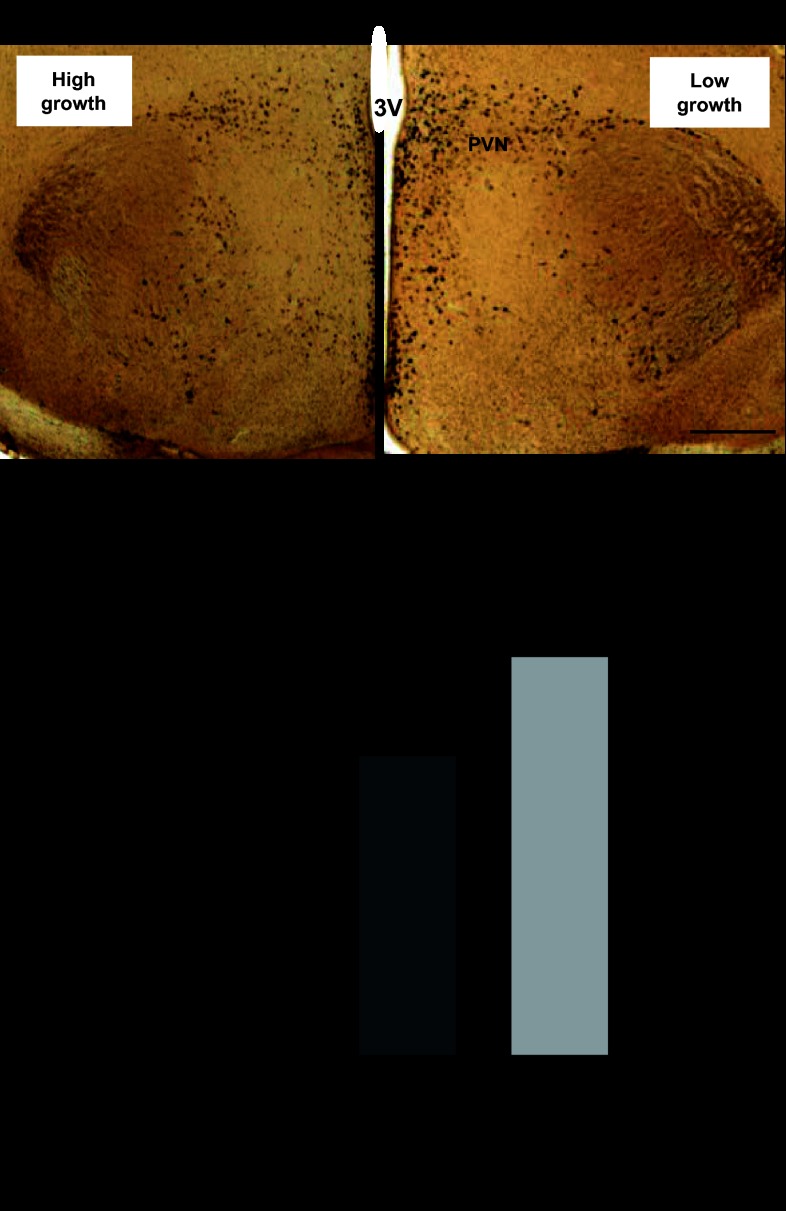
*A*: photomicrographs comparing CCKAR immunoreactivity in the hypothalamus of high-growth and low-growth haplotype chickens. Scale bar, 50 μm. *B*: total no. of CCKAR-immunoreactive cells observed using antibody CCKAR2F in the hypothalamus taken from 12-wk-old high-growth (*n* = 7) and low-growth haplotypes (*n* = 8); means ± SE. *P* value from ANOVA is shown.

The total number of CCKAR-immunoreactive cells quantified in the brain was significantly higher in the low-growth haplotype compared with the high-growth haplotype birds (Fig. 7).

**Fig. 8.**
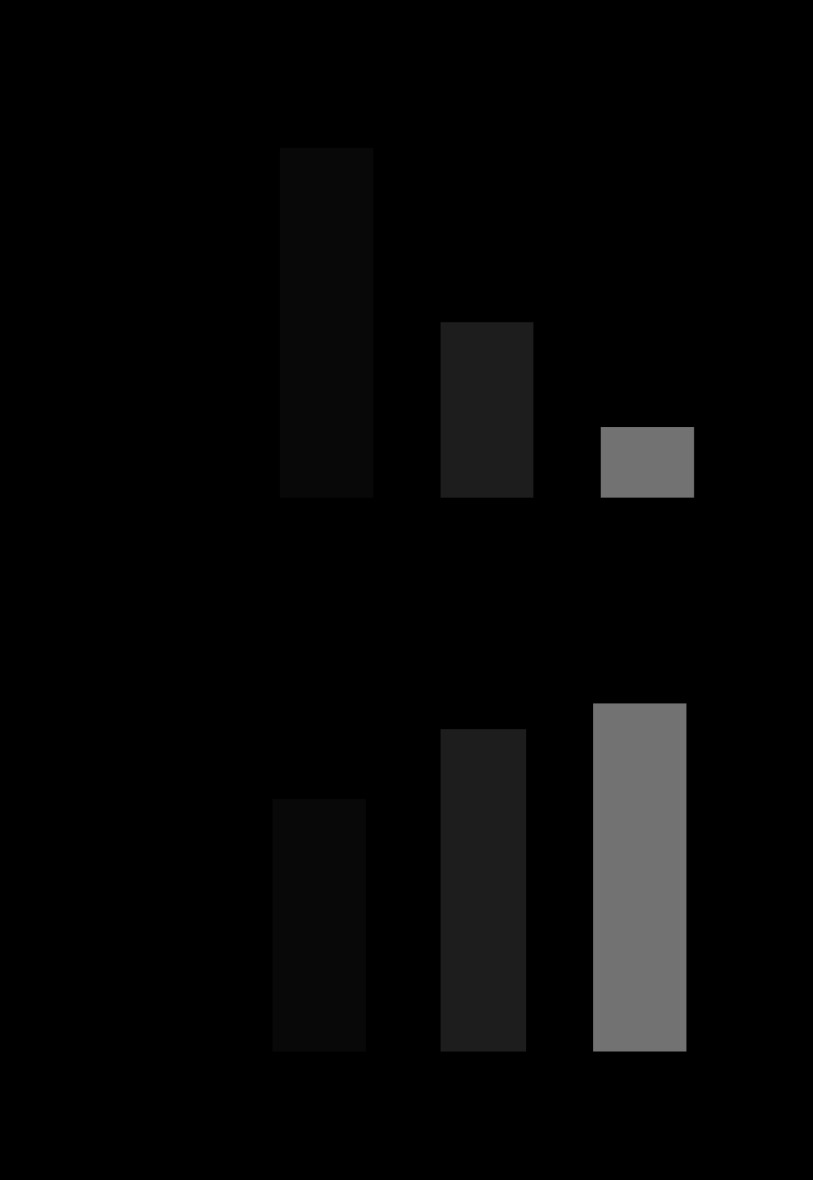
Expression of agouti-related protein (AGRP) and prooptiomelanocortin (POMC) mRNA in basal hypothalami taken from 12-wk-old homozygous high- or low-growth and heterozygote haplotypes (*n* = 8/group). *P* value from the ANOVA on log-transformed data is shown.

We anticipated that genes expressed in the feeding center neurons of the basal hypothalamus would also be altered because of the difference in CCK feedback through its receptor. We examined expression of AGRP and POMC genes that are factors driving orexigenic and anorectic behavior of birds and other vertebrates. AGRP expression in the high-growth haplotype was approximately fourfold higher than in the low-growth haplotype (Fig. 8). Although POMC expression in the high-growth haplotype tended to be less than in the low-growth haplotype, this was not significantly different between genotypes (Fig. 8).

## DISCUSSION

We have for the first time demonstrated that a change in a component of a satiety signaling system underlies the largest QTL for growth in chickens (32, 65), the first QTL for growth in which the underlying gene function has been understood in chickens. In mammals, the role of IGF-II in pig growth (72) and the double muscling caused by myostatin in breeds of cattle (26) are the only examples where function has been elucidated. The CCK system has been investigated as a cause of increased appetite of rapid growing strains of chicken (60) with limited success but, it has now been proven that expression of the seven-transmembrane domain CCKAR G protein receptor is responsible for part of the difference in growth between strains. This QTL has been observed repeatedly (34, 47, 64, 71), and the coincidence of the CCKAR gene at the locus has been noted (54), and a gene 5 Mbp upstream, TBC1D1, was proposed as a candidate from a selective sweep study (57). However, the physiological basis of the locus has never been elucidated. The most significant marker in both the F8 and F16 AIL generations was ch4snp-131-132-4046-S-2, 1.56 Mbp downstream of the CCKAR locus. In an earlier study, the QTL explained 3.6% of the total variance (65) in the F2, and in this study the best marker explained just under 5% of the variance in the F8 and F16. This is somewhat less than the 15–30% explained by the IGF-II locus in pigs but is in line with many QTLs (72). Comparison of the F8 and F16 populations did not further define the location. There are genes in the vicinity of this SNP, notably KCNIP4, SLIT2, and LCORL, that have been associated with human stature, but none are obvious candidates for altering growth and food intake. Of the genes in the loci that we did measure, we saw no difference in expression with genotype. In a whole genome study of egg weight, a trait correlated with body weight, the most significant SNP in 1-Mbp windows was at position 78,775,527, with a SNP of lesser effect at 78,724,797 (75). This may be the same locus underlying the difference in expression of CCKAR, and it confirms that the likely locus is at least 1.5 Mbp and perhaps 3 Mbp downstream of the CCKAR gene itself.

Evidence that the CCKAR locus is associated with growth and body size and that the genetic differences are not due to recent events came from the clear correlation between the frequency of the high-growth alleles at the CCKAR locus and the body weight at 10 wk of age in strains of chicken with markedly different growth rates. Because traditional heavy strains (e.g., White Sussex or Cornish game) are fixed for the high-growth alleles, this suggests that they were present in heavy breeds used to found modern broilers. These high-growth alleles are derived from fighting birds that tend to be larger and have greater musculature (77). Using marker CCKAR_MnlI, the correlation between the frequency of the high-growth allele in a strain and body weight suggests that 57% of the variance on the face of it can be explained by the genotype. Of course this is an overestimate, and we are not suggesting that this locus explains all of this variance. However, what it does indicate is that this haplotype has been selected or lost along with other loci related to growth and metabolism when selection has been practiced for a particular body conformation. In other words, the selection of an allele that increases food intake will have been accompanied by selection at other genetic loci that can influence utilization of the food for increased stature and muscle growth. The results suggest that the selection of the high-growth haplotype predates the development of heavy breeds for meat production (77), and is not a result of recent genetic selection. The fact that not all meat-type strains are fixed for the allele may reflect different founders for these lines or that the marker is not in complete linkage with the causative variation. The observation that from the list of the most significant markers in the region (Fig. 2) ch4snp-31-245-11611-S-1 and ch4snp-85-157-3063-S-2 were the only ones that showed any correlation with body weight suggests that the causative genetic loci may in fact be closer to the CCKAR gene itself than suggested by the analysis of the F8 or F16 population. Another possibility is that there are a number of independent genetic effects downstream of the CCKAR gene that occur in different strains that may interact with a region in the CCKAR gene locus.

The high-growth haplotype exhibited low expression of the CCKAR gene in almost all tissues examined. Also, in the hypothalamus, the number of CCKAR-immunoreactive cells was decreased, suggesting that the genotype also exhibits reduced protein expression. The difference in CCKAR immunoreactivity was not as pronounced as for mRNA in the hypothalamus, but this may reflect the limitation of counting visible cells rather than measuring protein content directly. The decreased expression of CCKAR in the high-growth haplotype appears to be functionally linked to our observation that the effect of intraperitoneally administered CCK in acutely reducing food intake was virtually abolished in that genotype compared with low-growth and heterozygote haplotypes. This suggests that the increased growth and body weight exhibited by the high-growth haplotype is at least partly due to a decreased level of CCK satiety signaling compared with the other genotypes. This may be reflected in altered meal patterns that promote weight gain. For example, in Otsuka-Long-Evans-Tokushima fatty (OLETF) rats (which have lost the promoter and exons 1 and 2 of the CCKAR gene by mutation) and in CCKAR-knockout mice, meal size is increased compared with control animals with normally functioning CCKARs (3). Analysis of the temporal structure of feeding behavior in chickens indicates the influence of satiety mechanisms (31, 70). Recent selection does not appear to have altered this parameter (30), which from our observation on the history of the CCKAR alleles would be expected. By contrast, comparison of fast-growing chickens did demonstrate that the amount eaten per meal was larger with fewer meals compared with light-bodied White Leghorns (5), and overall there were changes to the control of satiety between the strains, which the difference in CCKAR may underlie.

Although the influence of the high-growth haplotype on feeding behavior remains to be clarified, it is noteworthy that our results support an endogenous role for CCK signaling in body weight regulation in birds. In previous studies, it has been difficult to distinguish between true physiological and aversive effects of exogenously administered CCK (7, 12, 59, 69), and attempts to manipulate CCK receptors pharmacologically have been inconclusive (55).

Given that CCKAR expression was decreased in the high-growth haplotype in several central and peripheral tissues, it is difficult to be certain as to which tissue mediates the decreased feeding response to exogenous CCK, and it is indeed possible that several tissues are involved. Peripherally administered CCK appears to signal to the central nervous system via the vagus nerve in rats because the inhibitory feeding response to exogenous CCK is lost after vagotomy (68). However, in the chicken there are conflicting results, with vagotomy having no effect on preventing the reduction of food intake when CCK was administered intravenously (62) but having an effect when it was given intraperitoneally (13). The presence of Fos immediate early gene expression in the nucleus of the solitary tract after intraperitoneal CCK administration of quail is consistent with the activation of afferent pathways from the gut to the brain (7). Therefore, our observations of reduced expression of CCKAR in the high-growth haplotype in the duodenum and cecum could account for reduced signaling from CCK secreted into the gut to receptors on vagal afferents. The source of the ligand for the CCKARs we observed to be expressed in the central nervous system is less clear. In addition to mRNA expression in the hindbrain and basal hypothalamus, we found CCKAR-like immunoreactivity to be widely distributed in the hypothalamus, and its distribution pattern was broadly similar to that described in the rat for both mRNA and peptide (27, 44). At least some of the CCKAR-immunoreactive cells may be involved in the feeding response to CCK, and it is noteworthy that two of those nuclei, the PVN and PHN, also expressed significant Fos-like immunoreactivity after intraperitoneal injection of CCK in quail (7). In mammals, CCK reportedly does not cross the blood-brain barrier (51). However, there is the possibility that regions of the mediobasal hypothalamus and median eminence (where we observed dense CCKAR innervation) may be more directly accessible by circulating peptides (11), especially in light of the lack of effect of vagotomy combined with intravenous CCK (62). It is possible that the CCK ligand for central CCKARs is synthesized within neurons themselves in the chicken brain. Neuronal synthesis of CCK is long established in mammals (33, 73), and increased CCK-like immunoreactivity has been reported after meals (63). Thus CCK may also act as a centrally expressed neurotransmitter involved in the integration of visceral signals with the regulation of food intake. There is evidence for the expression of CCK in the avian brain (35). CCK-immunoreactive fibers were identified in the dorsal motor nucleus of the vagus and nucleus of the solitary tract in the pigeon (2), which were sites of Fos expression after CCK injection in the quail (7), and can be related to our observation of CCKAR mRNA expression in the chicken hindbrain. Immunoreactive CCK was also identified in the chicken hypothalamus (19), but although a recent distribution study of CCK mRNA in the zebra finch found widespread CCK expression, none was detected in the hypothalamus (40). Thus the extent of endogenous CCK synthesis in the chicken hypothalamus is currently uncertain. However, it is known that CCK reduces food intake in the chick after central administration (18, 69), and therefore, it is possible that the reduction we observed in hypothalamic CCKAR-immunoreactive cell numbers contributed to the reduced sensitivity of the feeding response to CCK in the high growth-haplotype.

We hypothesized that the reduced CCKAR expression observed in the hypothalamus would result in an elevation of orexigenic AGRP gene expression, and this was confirmed because the high-growth haplotype had a fourfold elevation of AGRP compared with the low-growth haplotype. Thus basal AGRP synthesis appears to be sensitive to the tone of CCK signaling, and its elevation is likely to contribute to the phenotype of the high-growth haplotype. Our finding is reminiscent of that of increased hypothalamic NPY synthesis in the OLETF rat (1), although the NPY cells concerned were in the dorsomedial nucleus rather than the arcuate nucleus. A link between melanocortin and CCK signaling has been suggested by the observation that sensitivity of the feeding response to CCK is reduced in melanocortin receptor-4 (MC4R)-knockout mice and that PVN neurons express the MC4R project to the nucleus of the solitary tract in the hindbrain (4). Given that AGRP is an MC4R antagonist, our findings are consistent with the direction of the change in sensitivity observed. It is uncertain as to how CCK signals are conveyed to AGRP neurons in the arcuate nucleus to influence their expression. The possibility for a direct effect of brain or circulating CCK is suggested by the observation of CCKAR (but not CCKBR) expression in the arcuate nucleus of the rat (27). We did not detect prominent CCKAR-like immunoreactivity in the infundibular nucleus in the present study, but it was one of the brain nuclei in which Fos expression was detected after intraperitoneal CCK injection in the quail (7). Therefore, it is possible that CCK signals are relayed to the infundibular nucleus via afferent visceral neural pathways.

CCKAR may be responsible for growth differences in other species, although function has not been proven. Polymorphisms, including one in a YY1 transcription binding site in the CCKAR promoter, were associated with growth in pigs (28, 29). It was suggested that snp.13.786.12116.S.3 might effect this transcription binding site in chickens, but no evidence was presented (54). The similarities between the pig and chicken loci are small, but the SNP does segregate with the high- and low-growth haplotypes in our population observed from our sequencing study. However, there are 37 differences that segregate with the genotypes, 12 in the promoter, and 15 in exons or in the immediate surrounding introns, which singularly or in combination could be responsible for differences in CCKAR expression. However, the strong association downstream of the locus suggests that a *cis*-acting effect, possibly an enhancer, is responsible. The observation that there is allele-specific expression, with the low-growth allele having the highest expression at ∼3.5 times that of the high-growth allele, strongly supports the hypothesis that the effect is due to a *cis* effect rather than, say, a transcription factor acting in *trans*. Without further testing, it is only speculative to ascribe function to any of these polymorphisms. Polymorphism in the human CCKAR promoter has been proposed to be responsible for differences in body fat (24), and the OLETF rat, which has a deletion in CCKAR (46), shows increased food intake. The absence of any effect on other genes at the locus suggests the effect is specific to CCKAR, but we cannot formally rule out effects on other genes in tissues we have not examined. The phenotype associated with reduced expression of CCKAR in high-growth chickens is similar to that postulated for humans, and an understanding of its molecular mechanism may be valuable to understand how this gene is regulated in humans. In mammals, CCK is thought of as controlling shorter-term food intake, such as meal size, whereas longer-term effects are mediated by factors such as leptin (50), which is lacking in birds (66). These results, along with the known mutants of CCKAR, clearly suggest that long-term effects on body weight can result from changes to the short-term feedback system. The CCK system may be worthy of attention in birds since in the absence of leptin it may play a more important role in the longer-term control of food intake and growth.

In this study, we have characterized the effects of reduced CCKAR expression on the central feeding center and on appetite. We also anticipate that there would be reduced secretion of bile and pancreatic polypeptides in the high-growth haplotype (39). None of these potential effects are likely to be conducive to more rapid growth; however, the potential to increase the transition of food through the digestive tract (15, 41) could improve growth. Further work using these genotypes will be required to characterize the endocrine and paracrine effects in the gut and how they promote growth.

In conclusion, we have for the first time elucidated the physiological basis of a QTL for growth in chickens, which affects appetite. The possession of segregating haplotypes has opened an opportunity to study how CCK feedback affects growth through appetite and food intake control. Food intake control has at times been controversial in avians, a clade that lacks leptin (20, 23, 66). This discovery also gives us an insight into the process of domestication and specialization of poultry strains and improves the prospect of deriving strategies to address some of the management problems resulting from correlated effects of selection for growth that result in the need to restrict heavy strains of poultry to permit normal reproduction (16).

## GRANTS

All authors were supported by the Biotechnology and Biological Sciences Research Council and the Roslin Institute through Institute Strategic Grant funding. C. A. Wardle and C. Hindar were supported by a Wellcome Trust vacation studentship to I. C. Dunn and S. L. Meddle, respectively. S. J. L. Ellison was supported by a vacation studentship from Pfizer to S. L. Meddle.

## DISCLOSURES

No conflicts of interest, financial or otherwise, are declared by the authors.

## AUTHOR CONTRIBUTIONS

I.C.D. and P.M.H. contributed to the conception and design of the research; I.C.D., P.M.H., P.W., C.A.W., V.B., C.H., G.W.R., D.W.B., S.J.E., and D.M.M. performed the experiments; I.C.D., P.M.H., S.M., P.W., C.A.W., A.L., V.B., C.H., G.W.R., D.W.B., S.J.E., and D.M.M. analyzed the data; I.C.D., P.M.H., S.M., P.W., C.A.W., A.L., S.J.E., and D.M.M. interpreted the results of the experiments; I.C.D., S.M., and V.B. prepared the figures; I.C.D. drafted the manuscript; I.C.D., P.M.H., S.M., P.W., C.A.W., V.B., and D.W.B. edited and revised the manuscript; I.C.D., P.M.H., S.M., P.W., C.A.W., A.L., V.B., C.H., G.W.R., D.W.B., S.J.E., and D.M.M. approved the final version of the manuscript.
